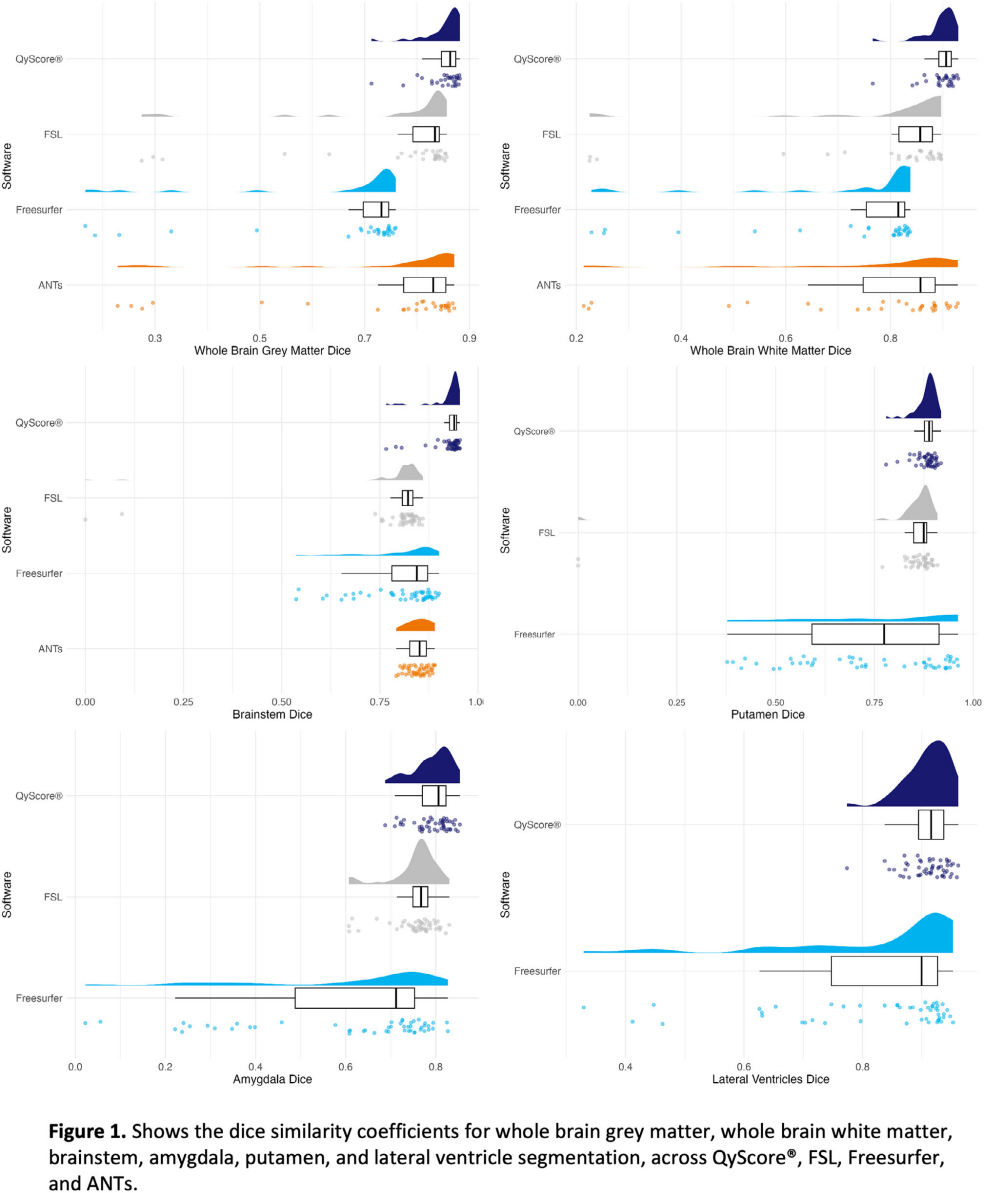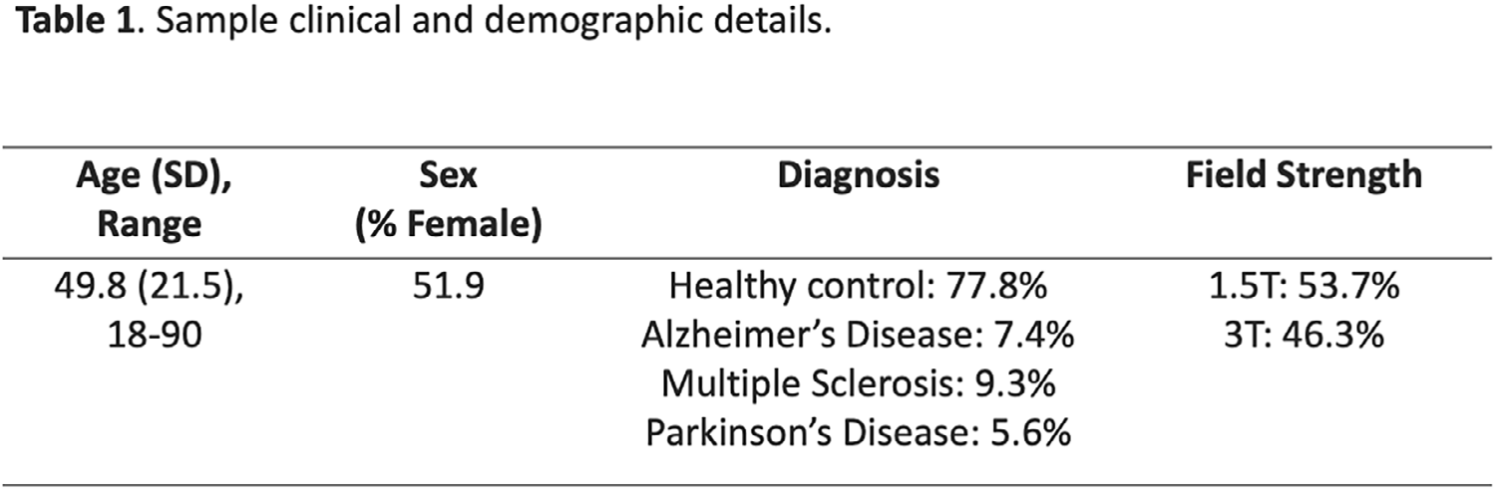# A Comparison of fully‐automated segmentation pipelines: QyScore® vs FreeSurfer vs FSL vs ANTs

**DOI:** 10.1002/alz70856_100760

**Published:** 2025-12-24

**Authors:** Luca Villa, Philippe Tran, Ayoub Gueddou, Mathilde Borrot, Nicolas Guizard, Elizabeth Gordon

**Affiliations:** ^1^ Qynapse, Paris, Île‐de‐France, France

## Abstract

**Background:**

The gold‐standard for brain segmentation is expert manual segmentation, which is time‐consuming and prone to low inter‐rater reliability. Work refining fully‐automated segmentation pipelines has resulted in many providing fast, reliable and accurate segmentations ‐ though performances can vary. QyScore^®^ is a medical imaging platform, designed for grey and white matter segmentation. We compared its performance to other state‐of‐the‐art segmentation pipelines, being FreeSurfer, FSL, and ANTs.

**Method:**

54 T1‐weighted images were used for manual and automatic segmentations of the whole brain grey matter (*n* = 30), whole brain white matter (*n* = 30), hippocampi (*n* = 48), amygdalae (*n* = 48), brainstem (*n* = 49), cerebellum (*n* = 49), caudate (*n* = 49), putamen (*n* = 49), thalamus (*n* = 49), globus pallidus (*n* = 49), and lateral ventricles (*n* = 49) (*Table 1*). Automated segmentations were produced by QyScore^®^ v1.13, FreeSurfer v7.4.1, FSL v6.0.6.2, and ANTs v2.5, using default parameters, followed by parameter optimization in the instance of preprocessing failures. Consensus manual segmentations, created by three expert neuroradiologists, were used as ground truth. Dice similarity coefficients (DSC) between consensus manual and automated segmentations were used to compare the accuracy of each segmentation pipeline.

**Result:**

QyScore^®^ was the best performing segmentation pipeline for whole brain grey matter, whole brain white matter, brainstem, amygdala, putamen, and lateral ventricles (Minimum‐Maximum DSC QyScore^®^:0.79‐0.93; ANTs:0.73‐0.85; FreeSurfer:0.61‐0.82; FSL:0.75‐0.83), *p_bonf_* <0.001; Figure 1. FSL performed the best for hippocampal segmentation (DSC=0.83), followed by QyScore® (DSC=0.82), and FreeSurfer (DSC=0.67), *p_bonf_* <0.05. There were no other significant differences in segmentation accuracy.

Initial automated preprocessing failed and required parameter optimization for 46.3% of subjects when using FreeSurfer, and 11.1% of subjects when using FSL. The preprocessing of one subject failed for FreeSurfer and FSL and were not recoverable without manual intervention. All subjects were preprocessed without error, using initial default parameters, by QyScore^®^ and ANTs.

**Conclusion:**

QyScore^®^ showed excellent segmentation accuracy across all brain regions, outperforming the other segmentation pipelines on the majority of regions. Alongside ANTs, it was the most robust pipeline in processing the wide variety of medical images without error. QyScore^®^'s high segmentation accuracy and robust pipeline demonstrate its utility in both healthcare and clinical trial settings, for supporting both clinical diagnosis and monitoring.